# Accessible and robust machine learning approaches to improve the opsin genotype-phenotype map

**DOI:** 10.1093/molbev/msag138

**Published:** 2026-06-16

**Authors:** Seth A Frazer, Todd H Oakley

**Affiliations:** Ecology, Evolution, and Marine Biology, University of California, Santa Barbara, CA 93106; Ecology, Evolution, and Marine Biology, University of California, Santa Barbara, CA 93106

**Keywords:** machine learning, regression, compiled database, genotype-phenotype map, predicting phenotypes, spectral sensitivity, color-vision, opsins, phylogenetics, cross-validation

## Abstract

Predicting phenotypes from genetic variation is a central challenge in biology. Here, machine learning (ML) offers great promise, but its use is often limited by poor accessibility, difficulty with interpretability, and a “data-cliff”—a gap between abundant sequences and scarce functional measurements. To develop more robust methods for genotype–phenotype prediction, an outstanding model system is opsin genes, visual pigments with extensive phenotypic information that strongly influence animal spectral sensitivity. Here, we advance ML characterization of the opsin genotype–phenotype map through four main contributions. First, we introduce the Opsin Phenotype Tool for Inference of Color Sensitivity, a user-friendly platform for predicting maximum wavelength sensitivity (λ_max_) from amino acid sequences, featuring integrated modules for SHapley Additive exPlanations and 3D structural mapping to reveal sequence-specific mechanistic drivers. Second, we show that encoding sequences with amino acid physicochemical properties improves predictive performance and interpretability over standard encoding methods and performs competitively with state-of-the-art protein language models, while retaining biological explainability. Finally, we present the Mine-N-Match pipeline, which systematically links published opsin sequences to compiled data on in vivo λ_max_ values, expanding genotype–phenotype coverage and improving prediction, especially for undersampled taxa. By integrating accessible software, biologically informed encoding, and data harmonization, our framework improves confidence, accuracy, and interpretability of genotype–phenotype predictions for animal opsins. An accurate genotype–phenotype map will allow simulating molecular evolution of function, reconstructing the history of visual phenotypes, designing functional proteins, and generating new hypotheses that can be tested with heterologous phenotyping.

## Introduction

Accurately relating phenotypes and genetic variation is a central challenge in evolutionary biology (Wagner 2011; Kemble et al. 2019; Nichol et al. 2019). A strong understanding of such genotype–phenotype maps will allow reconstructing historical shifts in gene function (Yokoyama et al. 2014; Storz 2016) and simulating or forecasting evolutionary pathways (de Visser and Krug 2014; Lässig et al. 2017). Accurate genotype–phenotype maps could transform fields including molecular evolution, medicine, agriculture, and protein engineering. Yet, despite recent progress in heterologous expression assays (Liénard et al. 2022; Smedley et al. 2022) and high-throughput mutational scanning (Fowler and Fields 2014; Inoue et al. 2021), empirical approaches remain costly and time-consuming, limiting their scalability, especially for broad comparative studies.

Artificial intelligence (AI) including machine learning (ML) provides a powerful complement to empirical work by learning predictive rules from genotype–phenotype datasets. These computational approaches accelerate discovery, reduce dependence on labor-intensive empirical assays, and generate testable hypotheses for experimental validation (Vamathevan et al. 2019; Hie et al. 2020; Frazer et al. 2021). For example, protein language models trained on millions of sequences capture structural and functional constraints (Rives et al. 2021; Lin et al. 2023), and large language models (LLMs) can simulate protein evolution and forecast mutational effects across deep timescales (Hayes et al. 2025). Despite this promise, barriers remain. First, most ML/AI tools require computational expertise, limiting accessibility to experimental biologists. Second, while predictive performance has advanced rapidly, connecting model features to specific residues or physicochemical properties remains challenging in many approaches, complicating biological interpretation. Third, a persistent “data-cliff”—a steep imbalance between abundant sequences and scarce functional measurements—limits the training of robust, generalizable models (Bhardwaj et al. 2025). Overcoming these barriers is essential if genotype–phenotype prediction is to become a reliable, widely used tool in molecular evolution.

To address these barriers, opsins provide an outstanding system for advancing genotype–phenotype prediction (Karasuyama et al. 2018; Adam et al. 2022; Frazer et al. 2024; Sela et al. 2024). In animals, these G-protein-coupled receptors form the molecular basis of vision and determine color sensitivity through their maximum wavelength of light absorption (λ_max_) (Govardovskii et al. 2000). Opsins are especially well-suited for methodological development because they combine massive sequence diversity with extensive phenotypic data from heterologous expression, in vivo measurements, and comparative studies (Terakita 2005; Yokoyama 2008; Hagen et al. 2023). Moreover, opsins have repeatedly diversified across metazoans, offering natural experiments in functional convergence and divergence (Porter et al. 2012; Ramirez et al. 2016; Vöcking et al. 2017). This combination of broad sequence sampling, rich functional characterization, and evolutionary repeatability makes opsins an excellent model for testing and refining ML approaches to genotype–phenotype mapping.

Here, we advance ML modeling of the opsin genotype–phenotype map through three main contributions. First, we introduce the *Opsin Phenotype Tool for Inference of Color Sensitivity* (OPTICS), a user-friendly platform that predicts maximum wavelength sensitivity (λ_max_) from amino acid sequences. To begin bridging the gap between sequence prediction and biophysical mechanism, OPTICS integrates SHapley Additive exPlanations (SHAP) (Lundberg and Lee 2017) and 3D structural mapping, offering powerful methods for local interpretability by assigning specific values of phenotypic contributions to individual residues and visualizing their position in the protein in three-dimensional space. Second, we show that encoding sequences with physicochemical properties of amino acids improves predictive accuracy and allows better mechanistic interpretability, while also performing comparably to complex protein language models. Finally, we present the Mine-N-Match (MNM) pipeline, which systematically links published opsin sequences to compile in vivo λ_max_ data, expanding genotype–phenotype coverage and improving prediction, especially for invertebrate opsins with sparse heterologous expression data. Together, these advances establish a methodological foundation for predictive molecular evolution using opsins as a model system—opening the door to simulating molecular evolution, reconstructing ancestral visual phenotypes, and generating testable hypotheses for experimental validation, while enabling direct between molecular evolution and visual ecological function in diverse light environments.

## Methods

### OPTICS: an accessible ML tool for predicting opsin spectral sensitivity

To improve the accessibility of our ML models, we developed the Opsin Phenotype Tool for Inference of Color Sensitivity (OPTICS), a comprehensive and user-friendly platform for predicting and interpreting opsin λ_max_ values from unaligned amino acid sequences. OPTICS is available as a command-line tool, graphical-user-interface (GUI), and a web application under the Galaxy system. The GUI integrates four distinct analytical pipelines: (1) Standard Predictions, (2) SHAP Interpretation, (3) Structure SHAP Mapping, and (4) Structure Annotations (all modules described in more detail below). Additionally, the GUI provides hyperlinked execution logs for seamless navigation of results. Across these modules, OPTICS provides users eleven best-performing ML models, trained on 11 different datasets, which can use either one-hot encoding (scoring each amino acid at each position as either present or absent) or an “amino-acid property” (aa-property) encoding scheme described herein. For prediction, OPTICS aligns the user's query sequence to the training alignment of the selected model using MAFFT (Katoh and Standley 2013).

In addition, OPTICS incorporates optional bootstrapping and BLASTp (Camacho et al. 2009) analyses. Bootstrapping (described in more detail below) quantifies uncertainty, including box plots, colored to reflect the median predicted λ_max_ using the corresponding hex-code color value. The BLASTp analysis compares query sequences against the training dataset of the selected model. Site numbering in BLASTp reports can be standardized to a chosen reference sequence (e.g. bovine or squid rhodopsin) or a custom one (see Methods S1 for implementation details and availability). OPTICS is implemented in Python, with core functionality built using scikit-learn (Pedregosa et al. 2011) for model execution and BioPython (Cock et al. 2009) for sequence handling, along with dependencies such as MAFFT, BLAST+, and standard Python data-processing libraries.

### Assessing prediction stability with bootstrapping

To evaluate the stability of λ_max_ predictions and estimate confidence intervals (CIs), we implemented a bootstrapping pipeline. For each VPOD opsin data subset (Frazer et al. 2024), we generated 100 pseudoreplicated datasets by randomly resampling with replacement entire sequences and their associated λ_max_ value. For each pseudoreplicate, we trained a separate ML model for predicting λ_max_ for sequences input into OPTICS. We report mean and median predictions across replicates, using the 2.5th and 97.5th percentiles to construct CIs (see Methods S2). To illustrate the procedure, we applied bootstrapping to three example sequences with disparate λ_max_: a short-wave sensitive (SW) opsin (OX393528.1-9544021-9545976) from the Tobacco Hornworm (*Manduca sexta*), a rhodopsin (Rho; MN519143.1) from the Bluespotted Stingray (*Neotrygon kuhlii*), and a long-wave sensitive (LWS) opsin (MK983124.1) from the Corkwing Wrasse (*Symphodus melops*).

### Encoding amino acid physicochemical properties for ML model training

We compared traditional one-hot encoding (each amino acid scored as present or absent at each position) to a novel encoding based on 12 physicochemical properties (aa-properties; see Methods S3 and Table S1 for a more detailed justification of aa-property choices and actual values used). In the new approach, we represented each amino acid as continuous values of amino acid properties. Unlike one-hot encoding, which treats all unseen residues at a site as equivalent, aa-property encoding allows all models to use information for residues not present in the training set (Figure S1a/b). We used *deepBreaks* (Baghbanzadeh et al. 2023; Frazer et al. 2024) to test all possible aa-property combinations across six VPOD datasets. For each dataset, we trained models with 12 ML algorithms and recorded standard performance metrics. Under a null hypothesis of no change between encoding types, we next used Wilcoxon Signed-Rank Tests to compare aa-property and one-hot models based on cross-validation error for the whole dataset (WDS) and on the accuracy of predicting λ_max_ of mutants using models trained only on wild type (WT) data (Gommers et al. 2022; Damian Riina et al. 2023; Frazer et al. 2024). Finally, we built visualization tools to assess the site-wise contributions of specific aa-properties to λ_max_ predictions (Methods S3).

To evaluate the performance of our biologically interpretable feature engineering against state-of-the-art representation learning, we extracted sequence embeddings using the ESM-2 (esm2_t36_3B_UR50D) protein language model (Lin et al. 2023). Embeddings were extracted for all the opsin datasets from VPOD v1.2. Using a modified version of the deepBreaks pipeline (Frazer et al. 2024), we trained regression models (including GBR and XGBoost) on these high-dimensional ESM-2 embeddings and compared their predictive metrics (e.g. R^2^, MAE) to our models trained using aa-property encoding. All extraction code, Jupyter notebooks for model training, and resulting comparative model metrics for this benchmarking analysis are available in the VPOD GitHub repository.

### Expanding model interpretability with SHAP and 3D models

To move beyond global feature importance (the relative importance of individual features to a model) and provide sequence-specific mechanistic insights, we integrated SHAP (Lundberg and Lee 2017) into OPTICS. This feature operates in two primary modes. In “single” mode, OPTICS calculates the SHAP values for individual query sequences, quantifying how much each specific amino acid state or physicochemical property shifts the predicted λ_max_ away from the model's base value (average predicted λ_max_ of the decision-tree based models; e.g. XGB, GBR, and RF). In “comparison” mode, OPTICS performs pairwise comparisons between user-provided sequences, calculating the difference in SHAP values to attribute predicted spectral tuning shifts to specific amino acid differences between the two sequences. To allow for easy analysis of biological relevance, the pipeline automatically maps feature indices back to the target sequence numbering and/or a standardized reference sequence (e.g. Bovine Rhodopsin).

To facilitate spatial understanding of sequence features, we developed two structural visualization modules in OPTICS. The first module, “Structure SHAP Mapping,” projects the calculated SHAP importance values directly onto 3D protein structures. The tool parses the absolute SHAP values for a given sequence, aggregates them by residue position, normalizes the scores to a 0 to 100 scale, and replaces the B-factor (temperature factor) column of a target PDB file (e.g. Bovine Rhodopsin 1U19, or a user-provided custom PDB, including structures generated using AlphaFold). This effectively creates a 3D structural heatmap of ML feature importance.

The second module, “Structure Annotations,” allows users to visualize arbitrary, custom annotations (e.g. known tuning sites, mutational targets) without requiring coding expertise. Users provide a PDB file containing their target structure and a simple CSV file defining residue positions, colors, styles (e.g. spheres, sticks), and labels. OPTICS then generates automated execution scripts for popular molecular visualization software (PyMOL or ChimeraX) for rendering the 3D models. For an example input of this module see the file “optics_custom_annotations_ex.csv” in the “/examples” folder on the OPTICS GitHub.

### Assessing phylogenetic nonindependence in model evaluation

Because closely related sequences can inflate predictive performance when present in both training and testing sets, we evaluated the impact of phylogenetic nonindependence on model accuracy. We implemented a phylogenetically informed cross-validation scheme that assigns sequences to folds based on pairwise distances from a maximum likelihood opsin gene tree, generated with IQ-TREE (Minh et al. 2020), thereby reducing similarity between training and test partitions. Across a range of fold numbers and distance thresholds, this approach produced performance estimates broadly consistent with standard cross-validation, indicating that phylogenetic structure does not qualitatively alter model comparisons (see Methods S4). Given these results, and to maintain computational efficiency and usability, OPTICS uses standard cross-validation for model training and prediction.

### Optimizing model hyperparameters via grid search

To determine the best-fit hyperparameters for training ML models, we performed comprehensive grid searches (Pedregosa et al. 2011; Liashchynskyi and Liashchynskyi 2019; Baghbanzadeh et al. 2023) for the three algorithms (GBR, XGB, and RF), each applied to six primary VPOD data subsets (WDS, WT, Vert, Invert, WT-Vert, and T1), using both aa-property encoding and one-hot encoding strategies. For aa-property encoding, we first selected the top ten performing aa-property combinations (five by best R^2^, five by lowest MAE) from earlier combinatorial analysis. We recorded grid search-optimized R^2^ and MAE values and their corresponding hyperparameters, ultimately selecting the single best model algorithm and, for aa-property encoded models, the best aa-property combination and ML algorithm for each data subset (see Methods S5, Tables S2 and S3).

### Mine-N-match pipeline: linking genotypes to in vivo phenotypes

We developed the Mine-N-Match (MNM) pipeline to address the opsin “data-cliff,” where many more species have opsin sequences than associated λ_max_ measurements. MNM uses in vivo λ_max_ values compiled from prior studies, retrieves opsin sequences from the same species, predicts λ_max_ for those sequences with OPTICS, and expands the amount of sequence data with corresponding phenotypes when predictions match measurements. We first assembled *VPOD_in_vivo_v1.0* (Methods S6) from six published compilations and additional literature searches to harmonize *in-vivo* λ_max_ data from microspectrophotometry (Liebman et al. 1972; Bowmaker 1984), electroretinograms (Jacobs et al. 1996; Rocha et al. 2016), and related techniques. As a proof of concept, *VPOD_in_vivo_v1.0* is not comprehensive and additional λ_max_ measures can be added in future updates. Using the species list from *VPOD_in_vivo_v1.0,* we queried the NCBI taxonomy database (Schoch et al. 2020), corrected names with the Global Biodiversity Information Facility (GBIF) taxonomy when necessary (Lane 2003; Edwards 2004), and retrieved opsin coding sequences (nucleotide and protein) from NCBI (Sayers et al. 2025). To minimize the inclusion of nonvisual opsins from unspecific genome-wide selections, our automated NCBI queries incorporated an extensive list of explicit exclusion keywords (e.g. “melanopsin,” “OPN4,” “non-visual”). We supplemented these from other compiled sources to capture sequences absent from NCBI (Methods S6). Before the matching step, all retrieved candidate sequences were subjected to a BLASTp query against a curated reference database containing both visual and nonvisual opsins. Any sequence returning a top hit to a nonvisual opsin was strictly discarded. We then deployed the “whole-dataset,” aa-properties, model on OPTICS_v1.2 to predict λ_max_ for all candidate sequences and matched each predicted value to the closest in vivo λ_max_ measurement for each species in *VPOD_in_vivo_v1.0*. Matches were filtered to retain only the best match (absolute difference ≤10 nm) and to remove sequences already in VPOD or flagged as unlikely opsins by OPTICS BLASTp output. While we employ a strict 10 nm threshold here to serve as a conservative consistency filter, researchers utilizing the open-source MNM pipeline can easily adjust this parameter, or evaluate the unfiltered output files provided in our repository, to explore results using alternative stringencies. To track potential matching ambiguity, the pipeline automatically outputs a “candidate_opsin_count” for each match, recording the total number of candidate sequences for that species falling within the 10 nm threshold. We integrated these genotype–phenotype pairs into VPOD_v1.3, adding new “physiologically inferred” data subsets, each named with an “MNM” suffix in metadata and FASTA files. We trained models on these subsets and compared their performance to models trained on heterologously expressed opsins (see Methods S7).

To evaluate the fidelity of the MNM pipeline, we conducted a blind-retrieval validation test. We randomly selected and withheld 50 wild-type opsin sequences from the “whole-dataset” (specifically excluding any opsins with corresponding mutant data to avoid data leakage). We then trained a new OPTICS model on the remaining sequences. Treating the 50 true in vitro λ_max_ values as mock in vivo measurements, we ran the full MNM pipeline; querying NCBI for the respective species, predicting λ_max_ with the newly trained model, and filtering for best matches. We then evaluated the proportion of correctly recovered target sequences to quantify pipeline accuracy.

## Results

### OPTICS: an accessible ML tool for predicting opsin spectral sensitivity

**Figure 1:**
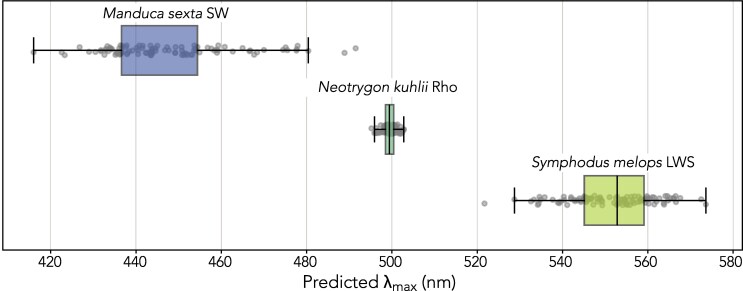
Box plot distributions of bootstrapped λ_max_ predictions illustrate the contrasting prediction confidence (distribution width) from the WDS bootstrap-ensemble when predicting the λ_max_ for a short-wave (SW) opsin (OX393528.1-9544021-9545976) from the Tobacco Hornworm (*Manduca sexta*) (95% CI width = 54 nm, SD = 14.7), a Rhodopsin (Rho; MN519143.1) from the Bluespotted Stingray (*Neotrygon kuhlii*) (95% CI width = 6.2 nm, SD = 1.6 nm), and long-wave sensitive (LWS) opsin (MK983124.1) from the Corkwing Wrasse (Symphodus melops)(95% CI width = 34.1 nm, SD = 10.0 nm). Note, the colors of the boxes automatically reflect the median predicted λ_max_ converted to the corresponding hex-code value. This method of uncertainty quantification and visualization formed an integral component for reporting OPTICS prediction uncertainty.

The Opsin Phenotype Tool for Inference of Color Sensitivity (OPTICS) enables rapid prediction of opsin spectral sensitivity (λ_max_) from protein sequences using ML models trained on genotype–phenotype data from the Visual Physiology Opsin Database (VPOD) (Frazer et al. 2024). OPTICS offers multiple pretrained models tailored to different taxonomic groups and allowing different sequence encoding strategies, including one-hot and amino acid property (aa-prop) encoding. In addition to λ_max_ prediction, OPTICS provides optional analyses for sequence interpretation, including BLASTp comparisons to training data, site-difference reports standardized to reference sequences, and bootstrap-based confidence estimates that can be visualized as distribution plots (Fig. 1). The tool outputs predicted λ_max_ values (mean, median, percentile-based CI, and SD) in TSV and Excel formats, along with metadata on the model, encoding method and a parameter log; optional outputs include BLASTp reports and bootstrap plots. OPTICS is freely available via GitHub and as a web application using the Galaxy Project platform (Galaxy Community 2024). Moreover, the standalone desktop GUI further democratizes these capabilities by uniting all OPTICS features—standard predictions, SHAP analysis, and structural mapping—under a single, user-friendly interface that handles multithreaded processing and provides hyperlinked execution logs for seamless navigation of results.

### Bootstrapping provides feedback on prediction stability and confidence

Bootstrapping produced quantitative CIs and allowed qualitative assessment of prediction distributions, as illustrated for the three example opsins (Fig. 1). The SW opsin from *M. sexta* exhibited a wide 95% CI (54 nm, SD = 14.7), the Rho from *N. kuhlii* had a conversely narrow CI (6.2 nm, SD = 1.6), and the LWS opsin from *S. melops* was of intermediate width (34.1 nm, SD = 10.0). In plots, box colors automatically reflect the median predicted λ_max_ converted to the corresponding hex-code value. This quantification and visualization of uncertainty using bootstrapping is an integral feature of OPTICS outputs.

### Encoding with physicochemical properties of amino acids enhances ML interpretability and predictive power

Across all datasets, models using aa-property encodings achieved higher R^2^ and lower error metrics than one-hot models (Table 1, Table S4). For example, in WDS, aa-property encoding improved R^2^ from 0.956 to 0.964 and reduced MAE by 18.5% (6.48 nm to 5.28 nm; *P* = 2.43 × 10^−14, Wilcoxon test). We also saw some improvement for WT models predicting mutant opsins, with MAE decreasing from 10.0 nm to 9.63 nm (*P* = 0.046, Wilcoxon test). Optimal aa-property sets differed by dataset, but hydrophilicity and hydrogen bonding properties appeared frequently. Although the magnitude of encoding improvement on mutants (MAE of 10.0 nm to 9.63 nm) is statistically significant, it is practically modest. Choice of well-fitted nonlinear algorithms (e.g. GBR, XGBoost) over others provides substantially larger gains than does the choice of encoding scheme; meaning the most benefit to prediction performance comes from simply selecting the appropriate algorithm class for your dataset (see the section “ML algorithm type contributes to the predictive power of ML models” from our previous work (Frazer et al. 2024) for a more detailed discussion of this point).

**Table 1:** Performance metrics across opsin subsets and top performing models using amino-acid property encoding.

Name	Data Subset Version	# Seqs	Top ML Algorithm	Top AA_Prop Combos	R^2a^	MAE [nm]^b^	MAPE [%]^b^	MSE^a^	RMSE^a^
Whole dataset (WDS)	*VPOD_wds_het_1.2*	1211	XGB	H1,P2,SCT	0.964	5.46	1.22	138	11.4
Wild-types (WT)	*VPOD_wt_het_1.2*	364	GBR	H2,H3,P1,NCI,PKA	0.939	8.01	1.73	181	13.0
Vertebrates (Vert)	*VPOD_vert_het_1.2*	1057	XGB	H2,H3,NCI,SCT,PKB	0.978	5.13	1.14	81.5	8.91
WT vertebrates (WT-Vert)	*VPOD_wt_vert_het_1.2*	319	GBR	H2,P2,V	0.979	4.73	1.01	56.2	7.14
Invertebrates (Invert)	*VPOD_inv_het_1.2*	155	GBR	H1,H3	0.831	13.4	2.86	515	21.2
Whole dataset MNM (WDS-mnm)	*VPOD_wds_het* *+* *vivo_1.0*	1724	XGB	H2,H3,NCI,MASS	0.959	6.23	1.32	138	11.6
Wild-types MNM (WT-mnm)	*VPOD_wt_het* *+* *vivo_1.0*	877	GBR	H2,P1,NCI,MASS,SASA,PKB	0.951	8.14	1.74	168	12.8
Vertebrates MNM (Vert-mnm)	*VPOD_vert_het* *+* *vivo_1.0*	1393	XGB	H3,P2,SCT,PKA,PKB	0.983	4.34	0.96	62.4	7.71
WT vertebrates MNM (WT-Vert-mnm)	*VPOD_wt_vert_het* *+* *vivo_1.0*	655	XGB	H2,H3,PKB	0.981	4.64	0.98	52.3	6.83
Invertebrates MNM (Invert-mnm)	*VPOD_inv_het* *+* *vivo_1.0*	331	GBR	H1,P1,SCT	0.896	13.0	2.81	473	20.7
Type-One Opsins (T1)	*Karyasuyama_T1_ops*	884	XGB	H3,P1,PKB	0.846	7.99	1.49	141	11.8

**Summary:** Machine learning (ML) models trained on opsin genotype–phenotype data using optimal sets of amino-acid properties are capable of accurately predicting λ_max_.

^a^R^2^, mean square error (MSE) or root mean square error (RMSE) are often interpreted as direct measures of comparing/analyzing model performance and used as training loss terms of the objective function—which measures how well the model fits the training data. One has to often balance between this and the regularization term, which controls the complexity of the model. Thus, a high performance is both simple and predictive; a tradeoff referred to as the “*bias-variance”* tradeoff. Additionally, note that the values presented here do not reflect performance metrics obtained with PW-CV; Table S5 provides all available information on Wild-Type model performance metrics under different PW-CV parameters.

^b^Mean absolute error (MAE) and mean absolute percent error (MAPE) are in relation to the absolute error λ_max_ predictions and interpreted in the same units of “nm.”

To evaluate whether our biologically informed feature engineering was competitive with state-of-the-art representation learning, we benchmarked our top-performing regression models (GBR, XGBoost) using sequence embeddings extracted from the ESM-2 protein language model (Lin et al. 2023) in place of our aa-property encodings. Models trained on ESM-2 embeddings yielded performance metrics that were similar to, but in all cases slightly worse than, models trained on our optimized combinations of physicochemical properties (Table 1, Table S5; ESM-2 WDS R^2^ = 0.961, MAE = 7.28 nm vs. AA-Prop WDS R^2^ = 0.964, MAE = 5.49 nm). Because models trained on embeddings fundamentally sacrifice site-wise mechanistic explainability, a core objective of the OPTICS framework, and require substantial additional computational overhead to embed every new query sequence prior to prediction, we retained physicochemical property encoding as the optimal balance of high predictive accuracy, computational efficiency, and biological interpretability.

**Figure 2:**
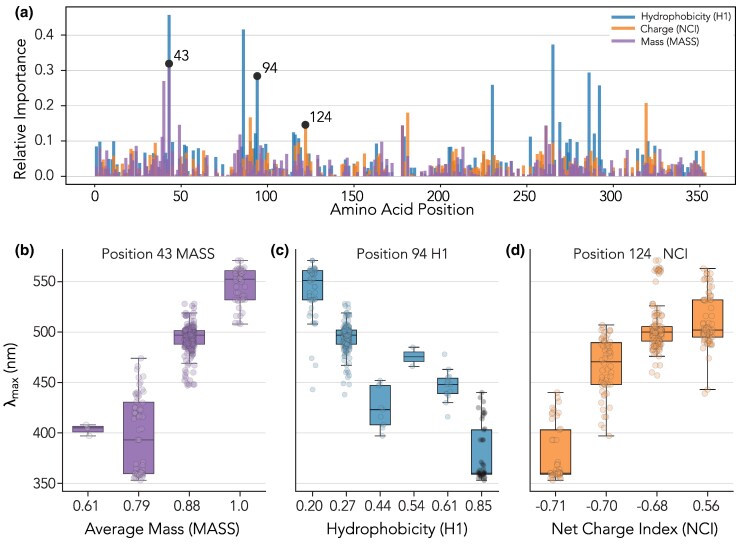
A WT model trained on the amino-acid property (aa-property) combination of hydrophobicity (H1), net charge index (NCI), and mass (MASS) reveals that properties such as amino-acid mass at site 43, hydrophobicity at site 94, and amino-acid charge at site 124 exerted relatively high influence on predictions of λ_max_. a) Bar graph of relative importance by position generated via the mean importance values from the “BayesianRidge,” “GBR,” “XGB,” and “RF” ML regression model trained on the WT-Vertebrate opsin dataset. We interpret positions with higher relative importance as having a larger effect or weight on λ_max_ prediction. Positions 43, 94, and 124 are highlighted with stars because they are among the highest scoring sites, show clear trends between target aa-property and associated λ_max_ distributions, and have all been previously characterized as functionally important to opsin phenotype and function. b to d) Distribution box plots for sites 43, 94, and 124, comparing the relationship between selected aa-property and associated range of λ_max_ values. The top distribution box plots provide a biologically interpretable visualization for the relationship between the measure of a physiochemical aa-property and associated range of λ_max_ values at a site of interest. At sites 43 and 124, increasing amino-acid mass and charge values display a positive correlation with true λ_max_ values, while increasing hydrophobicity is negatively correlated with λ_max_ at site 94. This ease of biological interpretation is lost when we simply visualize the relationship between amino-acids (as categories) and λ_max_. Note, all aa-properties are normalized, either −1 to 1 or 0 to 1 for ML training. Thus, these values do not reflect true units. For a more detailed explanation on how position importance scores are calculated for different models, refer to the “Interpretation” heading under the methods section of the deepBreaks publication (Baghbanzadeh et al. 2023).

We also used aa-property encoding to explore biological interpretability. Visualizations of site–property importance (Fig. 2a) showed that amino acid mass at site 43, hydrophobicity at site 94, and charge at site 124 strongly influenced predictions of λ_max_. Furthermore, at site 94 (Fig. 2c), we find higher hydrophobicity (H1) to be correlated with lower λ_max,_ while at site 124 (Fig. 2d) we find the net charge index (NCI) of amino acid to be positively correlated with λ_max_. These property–phenotype relationships would be less apparent in amino acid identity plots using standard one-hot encoding, as it treats amino acids as simple categorical objects, thereby obscuring the trends revealed when working with continuous aa-property values. This highlights an advantage of property-based visualizations for mechanistic insight.

### SHAP reveals sequence-specific and pairwise mechanistic drivers of phenotype predictions

**Figure 3:**
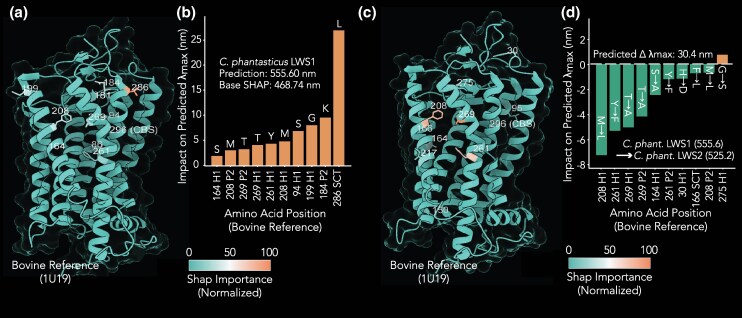
OPTICS integrated SHAP and structural mapping reveals mechanistic drivers of spectral tuning for single sequences and pairwise comparisons. a) Single-sequence SHAP explanation for the Long-Wave Sensitive 1 (LWS1) opsin of *C. phantasticus* (sequences provided as supplementary data, SD1). The bar plot displays the top sequence features—mapped to Bovine Rhodopsin reference numbering—and their specific physicochemical properties (e.g. Side Chain Type [SCT] at site 286, Polarizability [P2] at site 184) contributing to the predicted λ_max_ of 555.60 nm, relative to the model's base value of 468.74 nm. b) 3D structural mapping of the single-sequence SHAP values from (a) onto the Bovine Rhodopsin (1U19) reference structure. Normalized SHAP importance scores are visualized as a heatmap (cyan to orange), physically locating the most influential predictive residues within the transmembrane helices. c) Pairwise SHAP comparison illustrating the specific features driving a 30.4 nm predicted blue-shift between two paralogs, *C. phantasticus* LWS1 (555.6 nm) and LWS2 (525.2 nm). The plot quantifies how specific amino acid substitutions and their corresponding property changes (e.g. a shift from Methionine [M] to Leucine [L] at position 208 increasing hydrophobicity, [H1]) contribute additively to the difference in predicted phenotype. d) 3D structural mapping of the pairwise SHAP difference values from (c) onto the Bovine reference structure, highlighting the spatial distribution of the mutations responsible for the functional divergence between the LWS1 and LWS2 paralogs.

While global feature importance highlights universally critical tuning sites (e.g. sites 43, 94, 124), it cannot easily explain the specific contribution of individual sequence features to a predicted phenotype nor the reasons for predicted shifts in phenotype between two closely related sequences. Using our integrated SHAP pipeline, OPTICS successfully deconstructs individual λ_max_ predictions into additive feature contributions. For any given query sequence, the tool generates summary files and bar plots detailing how amino acid properties (e.g. a highly hydrophobic residue at a specific reference position) push the predicted λ_max_ higher or lower relative to the training dataset average (Fig. 3a). Furthermore, the pairwise comparison mode successfully isolates the exact feature differences driving predicted spectral shifts between any two sequences (Fig. 3c); a feature potentially most useful for understanding spectral tuning sites which drive differences in predicted λ_max_ between closely related opsins. By generating all-to-all difference matrices and pairwise SHAP comparison plots, the tool provides immediate, testable hypotheses regarding which specific mutations are responsible for observed or predicted functional differences in target visual systems. For example, when comparing the LWS1 and LWS2 paralogs of *C. phantasticus* (Sommer 2025; Fig. 3c; sequences provided as supplementary data, SD1), OPTICS explicitly identifies that a substitution at position 208 from Methionine (M) to Leucine (L), which increases site hydrophobicity (H1), is the primary driver of the 30.4 nm predicted blue-shift between the two sequences, followed by property shifts at sites 261 and 269.

The utility of the SHAP module as a hypothesis generating tool is further elevated when used in combination with the “SHAP structure mapping” module, allowing for three-dimensional visualizations of the sequence features, which impact individual phenotype prediction or which separate two sequences in predicted phenotype space (Fig. 3b/d). Referring back to the pairwise comparison of *C. phantasticus* paralogs, structural mapping quickly reveals how the potential tuning sites driving paralog divergence (such as sites 208, 261, and 269) are spatially distributed around the retinal binding pocket (Fig. 3d). Our structural mapping pipeline maps SHAP-derived importance scores onto the B-factor column of both the standard bovine rhodopsin (1U19) structure and user-provided PDB files, repurposing this field to encode residue-level importance rather than atomic displacement. Additionally, the custom annotation module automates visualization of user-defined sites of interest (e.g. residues surrounding the binding pocket, in combination with SHAP-highlighted sites), bypassing the need for molecular visualization scripting.

### Assessing the effects of phylogenetic nonindependence on model performance

To evaluate whether phylogenetic relatedness inflates performance estimates, we applied a phylogenetically informed cross-validation scheme that partitions sequences based on evolutionary distance (see Methods; Methods S4). We found that increasing the stringency of phylogenetic separation led to modest declines in performance metrics (e.g. R^2^), reflecting reduced similarity between training and test data (Figure S2, Table S6). These effects did not qualitatively alter comparisons among models, encoding strategies, or algorithm classes. In particular, aa-property encoding continued to outperform one-hot encoding, and tree-based methods (e.g. GBR, XGBoost) remained the top-performing algorithms under phylogenetically informed partitioning. Together, these results indicate that while phylogenetic structure influences absolute performance estimates, it does not change the relative conclusions of our analyses. Given this, and to maintain computational efficiency and accessibility, OPTICS uses standard cross-validation for model training and prediction.

### Hyperparameter optimization via grid search identifies dataset-dependent parameters, enhancing ML predictive power

For models trained with one-hot encoded sequences, our grid search (gs) pipeline identified the specific algorithm (among GBR, XGB, and RF) and its corresponding hyperparameters that yielded the highest R^2^ for each individual dataset. While the best-performing algorithm varied depending on the dataset, the optimization process successfully pinpointed configurations maximizing predictive accuracy based on R^2^. The optimized hyperparameters and the resulting top R^2^ scores for the best-performing algorithm identified for each dataset are detailed in Table S2.

For models trained using aa-property encoded sequences, our grid search pipeline identified the single best-performing combination of aa-properties and ML algorithm hyperparameters for each dataset, based on maximizing the postgrid-search R^2^ value. The specific aa-prop combination selected, the best-performing algorithm with its optimized hyperparameters, and the corresponding peak R^2^ values achieved for each dataset are documented in Table S3.

### ML can help link genotype data to in vivo measurements of phenotype

*VPOD_in_vivo_v1.0* contains 3810 unique in vivo λ_max_ measurements across 1397 species from nine phyla. From those species, we mined 2565 candidate visual opsin sequences from 329 species (split between NCBI and supplementary sources). The MNM pipeline matched predictions from these sequences to physiological λ_max_ values, yielding 852 initial matches, filtering for best match per species and removing duplicates. After removing poor matches (>10 nm difference between prediction and in vivo measurement), we retained 526 genotype–phenotype links for use in subsequent model training.

To quantify the risk of false matches caused by multiple viable candidates, we calculated the average number of opsins falling within the 10 nm threshold per in vivo target. On average, successful matches had 2.11 candidate opsins; however, this figure was driven primarily by a few outlier teleosts and insect species possessing 10 to 18 potential candidates, suggesting that the vast majority of matches face minimal ambiguity. Furthermore, our blind-retrieval test demonstrated the high fidelity of the MNM pipeline. Out of 50 withheld wild-type sequences processed as mock in vivo targets, the pipeline successfully retrieved and correctly matched 47 sequences (94% accuracy). The three incorrect matches were entirely driven by incorrect paralog assignment within species containing multiple opsins with nearly identical λ_max_ values. In one case, two paralogs with true λ_max_ values of 561 and 562 nm were both predicted at 561.3 nm, resulting in an essentially randomly swapped assignment. The third mismatch similarly involved a sequence incorrectly matched over the correct paralog. The two opsins had predicted λ_max_ which differed by only 3.9 nm (490.8 and 494.7 nm) and were forced to match a single target λ_max_ value (491 nm).

### Harmonizing in vivo and in vitro data improves prediction of spectral sensitivity for undersampled opsin families

We integrated the 526 matches into *VPOD_v1.3*, creating five new data subsets that merge heterologous data and matched data (WDS-mnm, WT-mnm, Vert-mnm, WT-Vert-mnm, Invert-mnm). This addition doubled the number of wildtype genotype–phenotype links, as illustrated by the wild-type (WT) (364 to 877) and invertebrate (Invert) (155 to 331) datasets. Models trained on MNM-augmented subsets consistently outperformed those trained on heterologous-only data. For example, a gs-optimized aa-property model on Invert-mnm achieved R^2^ = 0.901, MAE = 13.2 nm, compared with R^2^ = 0.839, MAE = 13.1 nm for heterologous-only Invert. The WT-mnm model likewise improved, if only slightly (R^2^ = 0.949 vs. 0.939; MAE = 7.17 nm vs. 8.08 nm). Gains were especially strong for understudied invertebrate opsins, suggesting that incorporating inferred in vivo data improves generalization and accuracy (Table 1; Tables S2 to S6). We deployed these new MNM-trained models, plus bootstrap versions, as part of the OPTICS_v1.3 public release on GitHub.

## Discussion

Accurately predicting protein function from sequence remains a central challenge in molecular evolution. We advance this goal using opsin visual pigments by integrating interpretable ML and systematic data harmonization. Our contributions include the following: (1) OPTICS, an accessible and flexible tool to predict λ_max_; (2) encoding based on amino acid properties that improve accuracy and generalizability over one-hot encoding; (3) bootstrapping and to better understand the uncertainties of model predictions; and (4) the *Mine-N-Match* (MNM) pipeline, which bridges the “data-cliff” between sequence-rich and phenotype-poor datasets. Together, these innovations provide a framework for more accurate genotype–phenotype prediction and make these capabilities broadly available for visual ecology and molecular evolution.

### Democratizing phenotype prediction

Powerful ML models and structural biology tools often require specialized computational expertise, limiting their reach. OPTICS removes this barrier by offering command-line, GUI, and web-based interfaces that predict λ_max_ directly from amino acid sequences, while requiring minimal bioinformatics background. The tool packages pretrained models across data subsets and encoding strategies, automates alignment insertion, performs bootstrap analysis for CIs, and generates SHAP explanations of phenotype predictions which it can then map onto 3D structures. This accessibility enables a wider community, especially visual ecologists and molecular evolutionists, to seamlessly incorporate predictive modeling and structural visualization into their research workflows.

Despite the broad utility of these ML models, caveats exist. The models predict λ_max,_ a central but incomplete descriptor of spectral sensitivity. λ_max_ does not capture activation kinetics or in vivo modifiers like screening pigments (Arikawa and Stavenga 1997), oil-droplets (Das et al. 1999; Hart and Vorobyev 2005; Toomey et al. 2015; Toomey and Corbo 2017), or chromophore shifts (Buczyłko et al. 1996; Terakita 2005; Sekharan and Morokuma 2011). The tool will also return predictions for any sequence, including nonfunctional or partial opsins, which may yield unreliable results, especially if missing data involve key tuning sites. While OPTICS alerts users when sequence identity to known opsins is low, future versions could incorporate automated filtering of nonopsins and pseudogenes. While we cannot state a specific “cut-off” for the reliability of predictions, as with any ML tool, predictions for highly divergent sequences require caution. Thus, such features as bootstrap intervals and BLASTp provide critical feedback for individual assessments of prediction reliability.

### Advancing ML genotype–phenotype links: confidence and interpretability

Reliable genotype–phenotype prediction from biological sequences requires three pillars: robust confidence estimates, evaluation methods that account for evolutionary relatedness, and biologically grounded interpretability. CIs provide important context for the reliability of predictions. Herein, bootstrapping demonstrated substantial variance in confidence across different opsins (Fig. 1); reflecting differences in how well-sampled are different regions of genotype–phenotype space. Without CIs, predictions from sparsely sampled paralogs could appear as certain as those for conserved, highly sampled genes, like vertebrate rhodopsins.

We found model interpretability to benefit from encoding strategies that reflect amino acid properties. One-hot encoding, while common, treats each amino acid as an unrelated category and fails to capture similarities based on shared physicochemical properties. By contrast, our amino acid property encoding improves both predictive accuracy and generalization to mutant sequences (Table 1, Table S4; Figure S2a and b), likely because it allows the model to exploit biologically relevant relationships, even for amino acids absent from the training set, and because amino acid properties may contain more information about changes to opsin tuning sites. Conversely, while highly parameterized protein language models (like ESM-2) offer powerful sequence representations (Brandes et al. 2023; Lin et al. 2023; Hayes et al. 2025), our benchmarking revealed that they provided no distinct predictive advantage over our optimized physicochemical property combinations for this specific task (Table 1, Table S5). Crucially, aa-property encoding allows the model to exploit biologically relevant relationships while explicitly preserving site-wise explainability; an attribute lost when sequences are transformed into high-dimensional embedding space.

Visualizations of site-wise importance from our aa-prop models link predictive patterns to known or candidate spectral tuning mechanisms. For example, our models identified hydrophobicity at site 94, a position in the binding pocket near the counterion at site 113 and the Schiff base linkage at site 296, as negatively correlated with λ_max_, consistent with the role of hydrophobic interactions in stabilizing the chromophore binding pocket (Takahashi and Ebrey 2003; Chinen et al. 2005; Tsukamoto et al. 2017). Similarly, charge at site 124, another known tuning site (Zheng et al. 2015; Castiglione and Chang 2018), showed a positive correlation with λ_max_. Such property–phenotype relationships provide direct, mechanistic hypotheses for experimental testing, and future work could also integrate epistasis or multiproperty effects to further enhance interpretability. That said, it is important to note that several properties are likely correlated (e.g. hydrophobicity, volume, SASA), which could complicate interpretation of aa-property feature importance. Future iterations of this work could seek to address this by applying an additional correlation filter when using combinatorial analysis to find the optimal aa-property combinations for a given dataset.

While global visualizations of site-wise importance provide a broad overview of tuning mechanisms, such as identifying the general role of hydrophobicity at site 94 or charge at site 124 in the binding pocket, they fail to explain the specific evolutionary trajectory of individual sequences. By integrating SHAP, OPTICS bridges this gap, providing local interpretability. SHAP values explicitly quantify how specific amino acid states in a given sequence contribute to its unique λ_max_, and the pairwise comparison tool isolates the exact features driving predicted functional divergence between two sequences. Crucially, OPTICS takes this interpretability a step further by mapping these SHAP values directly onto 3D protein structures. By converting ML feature importance into structural heatmaps, researchers can visually assess whether highly influential sites are structurally clustered, interacting directly with the chromophore, or exerting allosteric effects from the protein surface. As demonstrated with the SHAP comparison of *C. phantasticus* LWS paralogs, SHAP can highlight potential tuning sites driving paralog divergence (such as sites 208, 261, and 269; Fig. 3c) and structural mapping can quickly reveal how these potential tuning sites are spatially distributed around the retinal binding pocket (Fig. 3d). This seamless transition from raw sequence, to ML prediction, to biophysical structural context provides direct, mechanistic hypotheses for experimental testing (e.g. site-directed mutagenesis). Future work could also integrate interaction maps within these 3D SHAP maps to further enhance our understanding of the structural basis of spectral tuning.

### Bridging the data-cliff with mine-N-match

A persistent challenge in functional genomics is the data cliff, the steep disparity between the abundance of sequence data and the relative scarcity of experimentally characterized phenotypes. This imbalance limits our ability to fully use genomic information for predictive modeling of phenotypes. Opsins exemplify this challenge: while in vitro measurements of heterologously expressed sequences provide precise and controlled functional data, they are demanding technically, especially for invertebrate r-opsins, and thus limited in number. By contrast, in vivo measurements of spectral sensitivity (e.g. microspectrophotometry, electroretinograms) are more numerous across species but are rarely linked to the exact opsin sequences responsible.

The *Mine-N-Match* (MNM) pipeline addresses this gap by using predictions from ML as an informed bridge between unlinked in vivo phenotypes and available sequences. Importantly, MNM does not generate new phenotype values or sequences. Instead, it links independently measured in vivo λmax values and empirically derived opsin sequences from the same species using model predictions from OPTICS as a conservative consistency filter. MNM automates the retrieval of candidate opsin sequences for species with known in vivo λ_max,_ predicts λ_max_ for each candidate using OPTICS, and assigns the most likely sequence to a phenotype. Because this assignment requires close agreement between predicted and empirical λmax values, MNM acts as a conservative filtering step that prioritizes high-confidence matches while excluding ambiguous or potentially novel associations. This systematic approach accelerates what has traditionally been a slow, manual process of linking data scattered across public repositories and the literature. By combining automated NCBI queries, taxonomic reconciliation, and ML-based matching, MNM offers an efficient workflow for harmonizing heterogeneous datasets.

Integrating MNM-derived genotype–phenotype pairs into VPOD_v1.3 substantially expanded data coverage, doubling the number of wild-type opsins with linked phenotypes and nearly doubling invertebrate coverage—which are very undersampled in heterologous studies. We provide Figure S3 as an aid to visualize the distribution of λ_max_ phenotypes captured by VPOD_1.3, separated by vertebrate and invertebrate distributions. Models trained on these expanded datasets showed considerable gains, especially for invertebrate r-opsins, where predictive accuracy (R^2^) and error rates improved relative to models trained solely on in vitro data (Table 1). These results demonstrate that leveraging in vivo data—when genotype links are inferred by robust ML methods—can meaningfully improve prediction accuracy and generalization in data-sparse regions of sequence space.

Although developed for opsins, the MNM strategy could be more broadly applicable to other molecular systems where phenotypes are measured independently of genotypes—such as enzymes with kinetic parameters from biochemical assays but no linked gene sequence, or receptors with pharmacological profiles but unsequenced sources. By pairing systematic sequence mining with predictive modeling, analogous pipelines could help bridge similar data-cliffs across diverse areas of biology, accelerating the integration of legacy phenotypic data into modern sequence-based frameworks.

While the results of the MNM pipeline are promising, it is important to note that success relies on the accuracy of OPTICS predictions to correctly link genotypes to in vivo phenotypes. Our blind-retrieval test demonstrated that the pipeline is highly robust, accurately recovering 94% (47/50) of targeted sequences. However, as highlighted by the test's three mismatches, incorrect links are still possible, especially for species with multiple opsin paralogs or natural variants having similar predicted λ_max_ values close to a target in vivo measurement. This fact presents a relevant challenge for pinpointing the exact opsin gene responsible for the physiological measurement (although this a potentially less prominent issue beyond certain arthropods and fish clades as opsin duplication is often touted as a primary mechanism of spectral sensitivity diversification; Rennison et al. 2012; Porath-Krause et al. 2016; Sharkey et al. 2023; Schott et al. 2024). Furthermore, the 1 to 4 nm prediction differences that caused paralog swapping in our blind test represent a level of fine-grained resolution not typically obtainable with in vivo measurements (e.g. ERG, MSP), which may also differ systematically from heterologous expression due to intraocular optical filtering, oil droplets, or screening pigments. Thus, while this paralog ambiguity represents an accepted level of uncertainty, it likely has minimal impact on the resulting model generalization. Furthermore, some taxa are known to co-express multiple opsins within the same photoreceptor, resulting in intermediate spectral sensitivities that cannot be cleanly matched to any single underlying sequence (Dalton et al. 2014; Isayama et al. 2014; Schott et al. 2024). Finally, it is important to note that our pipeline has the potential to overlook matches due to large-effect mutations not represented in the training set, reinforcing existing biases rather than capturing novel effects. We fully accept this as a limitation but wish to reiterate as the goal of our pipeline is to use the existing summary of opsin genotype–phenotype information (our models) as a mechanism to infer new connections and expand the existing catalog for those who are interested. Future iterations of this pipeline would benefit immensely from the integration of in situ hybridization expression data to help resolve these ambiguities and validate MNM-inferred links and future work could involve testing the validity of matches by expressing opsins heterologously and comparing the in vitro λ_max_ to the in vivo measurement it was linked to by MNM. Moreover, there were a rather large number of λ_max_ values from *VPOD_in_vivo_1.0* that were not linked to any opsin sequences. These could be a good target for future empirical work.

### Implications & future directions

The methods and tools developed here have immediate relevance for visual ecology, sensory biology, molecular evolution, and protein engineering. OPTICS and the expanded *VPOD_v1.3* database can accelerate characterization of visual systems across taxa, enabling large-scale comparative studies and deeper insight into how visual sensitivities adapt to diverse ecological niches. The principles behind PW-CV and amino acid property encoding are broadly transferable to predictive modeling of other protein functions.

Looking forward, this framework lays the ground for more ambitious explorations of genotype–phenotype landscapes. We envision extending this work into an integrated evolution simulation pipeline (Wittmann et al. 2021; Howard et al. 2025) designed to probe historical contingency and adaptive trajectories in molecular evolution. By simulating evolutionary paths under different varying selective pressures, this pipeline could reveal how different genetic starting points might constrain or expand accessible phenotypes (Upton et al. 2021; Murphy and Westerman 2022), identify repeatable mutational paths to the same phenotype, and show how these paths depend on genotype and selection. Such simulations could help illuminate the predictability of evolution, the role of epistasis in shaping adaptive landscapes, and the molecular underlying visual diversity, further enhancing the role of opsins as a model for broader principles of the molecular evolution of functional diversity.

## Supplementary Material

msag138_Supplementary_Data

## Data Availability

All methods, results, and analyses from this study and our previous publication are available in the GitHub repository *The Visual Physiology Opsin Database (VPOD)*: https://github.com/VisualPhysiologyDB/visual-physiology-opsin-db. *Opsin Phenotype Tool for Inference of Color Sensitivity* (OPTICS), is available at: https://github.com/VisualPhysiologyDB/optics. To ensure long-term reproducibility, all major releases of OPTICS and VPOD, including the exact training datasets, metadata, and models used herein, are versioned, archived, and freely available via Zenodo (VPOD_v1.3 DOI: 10.5281/zenodo.19051998; OPTICS_v1.3 DOI: 10.5281/zenodo.18955371). All data and code are released under the GNU General Public License v3.0 (Open Source Initiative–approved). These resources also support applications using the *deepBreaks* ML framework (Baghbanzadeh et al. 2023) and the VPOD database for opsin research (Frazer et al. 2024).
