# Supplementary material for: Accessible and robust machine learning approaches to improve the opsin genotype-phenotype map
Source: Mol Biol Evol. 2026 Jun 16;43(6):msag138. doi: 10.1093/molbev/msag138 (PMC13286010; doi:10.1093/molbev/msag138)
Supplement: msag138_Supplementary_Data [file msag138_supplementary_data.pdf]

Supplementary Material For:

Accessible and Robust Machine Learning Approaches to Improve the Opsin Genotype-Phenotype Map

Seth A. Frazer[1], Todd H. Oakley[1,*]

Affiliations:
[1]Ecology, Evolution, and Marine Biology, University of California, Santa Barbara, California 93106

ORCID and Email:
Seth A. Frazer
sethfrazer@ucsb.edu
https://orcid.org/0000-0002-3800-212X

Todd H. Oakley
oakley@ucsb.edu
https://orcid.org/0000-0002-4478-915X
*Correspondence to oakley@ucsb.edu

Supplementary Table 1. Table of Scaled Amino Acids and their corresponding Physiochemical Properties

	H1	H2	H3	V	P1	P2	SASA	NCI	MASS	PKA	PKB	[a]Side Chain Type
A	0.61	-0.09	0.50	0.19	0.62	0.11	0.44	-0.68	0.38	2.34	9.69	3
C	0.44	-0.25	0.50	0.31	0.42	0.31	0.55	-1.00	0.55	1.96	10.28	1
D	-0.17	1.00	1.00	0.27	1.00	0.26	0.60	-0.91	0.62	1.88	9.60	6
E	-0.08	1.00	1.00	0.43	0.95	0.37	0.70	-0.69	0.69	2.19	9.67	6
F	0.90	-0.72	0.50	0.79	0.40	0.71	0.84	-0.46	0.79	1.83	9.13	2
G	0.54	0.06	0.50	0.00	0.69	0.00	0.33	0.56	0.31	2.34	9.60	5
H	0.09	-0.09	1.00	0.54	0.80	0.56	0.76	-0.81	0.74	1.82	9.17	6
I	1.00	-0.50	0.50	0.64	0.40	0.45	0.68	-0.58	0.61	2.36	9.60	3
K	-0.47	1.00	0.50	0.69	0.87	0.54	0.85	-0.61	0.69	2.18	8.95	6
L	0.84	-0.50	0.50	0.64	0.38	0.45	0.73	-0.36	0.61	2.36	9.60	3
M	0.62	-0.34	0.50	0.65	0.44	0.54	0.76	-0.72	0.70	2.28	9.21	3
N	-0.10	0.69	1.00	0.40	0.89	0.33	0.62	-0.70	0.61	2.02	8.80	4
P	0.36	0.06	0.50	0.29	0.62	0.32	0.55	1.00	0.52	1.99	10.60	5
Q	-0.14	0.13	1.00	0.55	0.81	0.44	0.73	-0.38	0.69	2.17	9.13	4
R	-1.00	1.00	1.00	0.72	0.81	0.44	0.73	-0.38	0.84	2.17	9.04	6
S	0.20	0.16	1.00	0.20	0.71	0.15	0.49	-0.70	0.47	2.21	9.15	4
T	0.27	-0.06	1.00	0.35	0.66	0.26	0.57	-0.71	0.54	2.09	9.10	4
V	0.85	-0.41	0.50	0.49	0.45	0.34	0.62	-0.32	0.53	2.32	9.62	3
W	0.71	-1.00	0.75	1.00	0.42	1.00	1.00	-0.46	1.00	2.83	9.39	2
Y	0.43	-0.66	0.75	0.81	0.48	0.73	0.89	-0.56	0.88	2.20	9.11	2

[a]Side Chain Type (SCT) is ranked by reactivity: 1=Cleft-Cysteine, 2=Cleft-Aromatic, 3=Cleft-Aliphatic. 4=Cleft-polar uncharged, 5=Cleft-Pro & Gly, 6=All else

We selected twelve physicochemical properties of amino acids (aa-properties): hydrophobicity (H1), hydrophilicity (H2), hydrogen bonding (H3), polarity (P1), polarizability (P2), volume (V), net charge index of side chains (NCI), average mass of the amino-acid(MASS), solvent-accessible surface area (SASA), acid-dissociation constant (PkA), base-dissociation constant (PkB), and side-chain type (SCT). All properties, except for SCT, were normalized (0-1 or -1-1).

Supplementary Table 2. Grid-Search Optimized Parameters Across Opsin Subsets and Top Performing Models Using One-Hot Encoding.

Name	Top ML Algorithm	gs-R^2	gs-MAE	gs-MSE	gs-Optimized Model Params
Whole Dataset (WDS)	GBR	0.961	6.48	155	{'gbr__learning_rate': 0.2, 'gbr__max_depth': 3, 'gbr__max_features': 'sqrt', 'gbr__n_estimators': 500}
Wild-Types (WT)	XGB	0.921	8.69	232	{'xgb__gamma': 1.0, 'xgb__learning_rate': 0.1, 'xgb__max_depth': 3, 'xgb__n_estimators': 300, 'xgb__reg_alpha': 0, 'xgb__reg_lambda': 0}
Vertebrates (Vert)	XGB	0.976	6.05	92.4	{'xgb__gamma': 0, 'xgb__learning_rate': 0.1, 'xgb__max_depth': 3, 'xgb__n_estimators': 200, 'xgb__reg_alpha': 1.0, 'xgb__reg_lambda': 1.0}
WT Vertebrates (WT-Vert)	GBR	0.975	5.04	67.4	{'gbr__learning_rate': 0.2, 'gbr__max_depth': 3, 'gbr__max_features': None, 'gbr__n_estimators': 200}
Invertebrates (Invert)	BR	0.808	15.0	616	{'BayesianRidge__alpha_1': 0.01, 'BayesianRidge__alpha_2': 1e-06, 'BayesianRidge__compute_score': True, 'BayesianRidge__fit_intercept': True, 'BayesianRidge__lambda_1': 1e-06, 'BayesianRidge__lambda_2': 0.01}
Whole Dataset MNM (WDS-mnm)	XGB	0.957	6.57	165	{'xgb__colsample_bytree': 0.8, 'xgb__gamma': 0, 'xgb__learning_rate': 0.1, 'xgb__max_depth': 5, 'xgb__n_estimators': 200, 'xgb__reg_alpha': 1.0, 'xgb__reg_lambda': 0.1, 'xgb__subsample': 1.0}
Wild-Types MNM (WT-mnm)	GBR	0.943	7.88	195	{'gbr__learning_rate': 0.1, 'gbr__max_depth': 5, 'gbr__max_features': 'sqrt', 'gbr__n_estimators': 500}
Vertebrates MNM (Vert-mnm)	XGB	0.980	4.75	73.3	{'xgb__colsample_bytree': 0.8, 'xgb__gamma': 1.0, 'xgb__learning_rate': 0.2, 'xgb__max_depth': 5, 'xgb__n_estimators': 100, 'xgb__reg_alpha': 0.1, 'xgb__reg_lambda': 1.0, 'xgb__subsample': 1.0}
WT Vertebrates MNM (WT-Vert-mnm)	XGB	0.980	4.43	52.8	{'xgb__colsample_bytree': 1.0, 'xgb__gamma': 0.1, 'xgb__learning_rate': 0.2, 'xgb__max_depth': 5, 'xgb__n_estimators': 100, 'xgb__reg_alpha': 0.1, 'xgb__reg_lambda': 1.0, 'xgb__subsample': 1.0}
Invertebrates MNM (Invert-mnm)	GBR	0.888	13.6	513	{'gbr__learning_rate': 0.1, 'gbr__max_depth': 3, 'gbr__max_features': 'sqrt', 'gbr__n_estimators': 200}
Type-One Opsins (T1)	XGB	0.820	9.03	166	{'xgb__colsample_bytree': 0.8, 'xgb__gamma': 0, 'xgb__learning_rate': 0.2, 'xgb__max_depth': 3, 'xgb__n_estimators': 200, 'xgb__reg_alpha': 1.0, 'xgb__reg_lambda': 0, 'xgb__subsample': 0.8}

**gs = grid-search*

Supplementary Table 3. Grid-Search Optimized Parameters Across Opsin Subsets and Top Performing Models Using Amino-Acid Property Encoding.

Name	Top ML Algorithm	gs-R^2	gs-MAE	gs-MSE	gs-Optimized Model Params
Whole Dataset (WDS)	XGB	0.970	5.33	112	{'xgb__colsample_bytree': 0.8, 'xgb__gamma': 0, 'xgb__learning_rate': 0.2, 'xgb__max_depth': 5, 'xgb__n_estimators': 100, 'xgb__reg_alpha': 0.1, 'xgb__reg_lambda': 0.1, 'xgb__subsample': 1.0}
Wild-Types (WT)	GBR	0.939	8.08	180	{'gbr__learning_rate': 0.1, 'gbr__max_depth': 3, 'gbr__max_features': 'log2', 'gbr__n_estimators': 500}
Vertebrates (Vert)	XGB	0.981	4.95	72.0	{'xgb__colsample_bytree': 1.0, 'xgb__gamma': 0, 'xgb__learning_rate': 0.1, 'xgb__max_depth': 5, 'xgb__n_estimators': 200, 'xgb__reg_alpha': 0.1, 'xgb__reg_lambda': 0.1, 'xgb__subsample': 1.0}
WT Vertebrates (WT-Vert)	GBR	0.980	4.60	54.7	{'gbr__learning_rate': 0.1, 'gbr__max_depth': 3, 'gbr__max_features': None, 'gbr__n_estimators': 300}
Invertebrates (Invert)	GBR	0.839	13.1	498	{'gbr__learning_rate': 0.01, 'gbr__max_depth': 3, 'gbr__max_features': None, 'gbr__n_estimators': 500}
Whole Dataset MNM (WDS-mnm)	XGB	0.965	5.91	133	{'xgb__colsample_bytree': 0.8, 'xgb__gamma': 1.0, 'xgb__learning_rate': 0.1, 'xgb__max_depth': 5, 'xgb__n_estimators': 300, 'xgb__reg_alpha': 0, 'xgb__reg_lambda': 0, 'xgb__subsample': 1.0}
Wild-Types MNM (WT-mnm)	GBR	0.949	7.17	175	{'gbr__learning_rate': 0.1, 'gbr__max_depth': 3, 'gbr__max_features': 'sqrt', 'gbr__n_estimators': 800}
Vertebrates MNM (Vert-mnm)	XGB	0.985	4.11	55.9	{'xgb__colsample_bytree': 0.8, 'xgb__gamma': 1.0, 'xgb__learning_rate': 0.1, 'xgb__max_depth': 5, 'xgb__n_estimators': 300, 'xgb__reg_alpha': 0, 'xgb__reg_lambda': 0.1, 'xgb__subsample': 0.8}
WT Vertebrates MNM (WT-Vert-mnm)	XGB	0.984	4.15	43.0	{'xgb__colsample_bytree': 0.8, 'xgb__gamma': 0, 'xgb__learning_rate': 0.1, 'xgb__max_depth': 5, 'xgb__n_estimators': 200, 'xgb__reg_alpha': 1.0, 'xgb__reg_lambda': 1.0, 'xgb__subsample': 0.8}
Invertebrates MNM (Invert-mnm)	GBR	0.900	13.2	456	{'gbr__learning_rate': 0.01, 'gbr__max_depth': 3, 'gbr__max_features': 'None', 'gbr__n_estimators': 500}
Type-One Opsins (T1)	XGB	0.869	7.55	121	{'xgb__colsample_bytree': 0.8, 'xgb__gamma': 0.1, 'xgb__learning_rate': 0.1, 'xgb__max_depth': 5, 'xgb__n_estimators': 300, 'xgb__reg_alpha': 1.0, 'xgb__reg_lambda': 1.0, 'xgb__subsample': 0.8}

**gs = grid-search*

Supplementary Table 4. Performance Metrics Across Opsin Subsets and Top Performing Models Using One-Hot Encoding.

Name	Data Subset Version	# Seqs	Top ML Algorithm	[b]R^2	[a]MAE [nm]	[a]MAPE [%]	[b]MSE	[b]RMSE
Whole Dataset (WDS)	*VPOD_wds_het_1.2*	1211	GBR	0.956	7.31	1.69	157	12.4
Wild-Types (WT)	*VPOD_wt_het_1.2*	364	GBR	0.912	9.43	2.04	257	15.4
Vertebrates (Vert)	*VPOD_vert_het_1.2*	1057	XGB	0.971	6.02	1.26	110	10.3
WT Vertebrates (WT-Vert)	*VPOD_wt_vert_het_1.2*	319	GBR	0.973	5.22	1.14	72.6	8.08
Invertebrates (Invert)	*VPOD_inv_het_1.2*	155	BR	0.808	15.0	3.27	616	23.4
Whole Dataset MNM (WDS-mnm)	*VPOD_wds_het+vivo_1.0*	1724	XGB	0.953	6.55	1.45	178	13.1
Wild-Types MNM (WT-mnm)	*VPOD_wt_het+vivo_1.0*	877	GBR	0.932	9.03	1.94	231	15.0
Vertebrates MNM (Vert-mnm)	*VPOD_vert_het+vivo_1.0*	1393	XGB	0.980	4.78	1.07	72.7	8.43
WT Vertebrates MNM (WT-Vert-mnm)	*VPOD_wt_vert_het+vivo_1.0*	655	XGB	0.983	4.64	0.98	46.8	6.77
Invertebrates MNM (Invert-mnm)	*VPOD_inv_het+vivo_1.0*	331	GBR	0.888	13.6	2.94	513	21.3
Type-One Opsins (T1)	*Karyasuyama_T1_ops*	884	XGB	0.803	9.05	1.69	180	13.3

[a]*Mean absolute error* (MAE) and *mean absolute percent error* (MAPE) are in relation to the absolute error λ_{max} predictions and interpreted in the same units of 'nm'. [b]R^2, *mean square error* (MSE) or *root mean square error* (RMSE) are often interpreted as direct measures of comparing/analyzing model performance and used as training loss terms of the objective function - which measures how well the model fits the training data. One has to often balance between this and the regularization term, which controls the complexity of the model. Thus, a high performance is both simple and predictive; a tradeoff referred to as the '*bias-variance'* tradeoff.

Supplementary Table 5. Performance Metrics Across Opsin Subsets and Top Performing Models Using ESM-2 Embeddings

Name	Data Subset Version	# Seqs	Top ML Algorithm	[b]R^2	[a]MAE [nm]	[a]MAPE [%]	[b]MSE	[b]RMSE
Whole Dataset (WDS)	*VPOD_wds_het_1.2*	1211	BR	0.961	7.28	1.66	147	12.1
Wild-Types (WT)	*VPOD_wt_het_1.2*	364	ET	0.888	10.6	2.36	337	17.8
Vertebrates (Vert)	*VPOD_vert_het_1.2*	1057	BR	0.977	5.99	1.38	87.5	9.24
WT Vertebrates (WT-Vert)	*VPOD_wt_vert_het_1.2*	319	HR	0.945	7.11	1.59	138	11.4
Invertebrates (Invert)	*VPOD_inv_het_1.2*	155	HR	0.813	14.5	3.22	644	23.0
Whole Dataset MNM (WDS-mnm)	*VPOD_wds_het+vivo_1.0*	1724	ET	0.946	8.47	1.90	205	14.2
Wild-Types MNM (WT-mnm)	*VPOD_wt_het+vivo_1.0*	877	ET	0.930	9.78	2.14	242	15.4
Vertebrates MNM (Vert-mnm)	*VPOD_vert_het+vivo_1.0*	1393	BR	0.978	5.82	1.32	79.2	8.87
WT Vertebrates MNM (WT-Vert-mnm)	*VPOD_wt_vert_het+vivo_1.0*	655	BR	0.964	6.34	1.36	97.6	9.43
Invertebrates MNM (Invert-mnm)	*VPOD_inv_het+vivo_1.0*	331	GBR	0.865	15.2	3.33	590	23.1

[a]*Mean absolute error* (MAE) and *mean absolute percent error* (MAPE) are in relation to the absolute error λ_{max} predictions and interpreted in the same units of 'nm'. [b]R^2, *mean square error* (MSE) or *root mean square error* (RMSE) are often interpreted as direct measures of comparing/analyzing model performance and used as training loss terms of the objective function - which measures how well the model fits the training data. One has to often balance between this and the regularization term, which controls the complexity of the model. Thus, a high performance is both simple and predictive; a tradeoff referred to as the '*bias-variance'* tradeoff.

Supplementary Table 6. *Phylogenetically-Weighted Cross-Validation* Performance Metrics For Grid-Search Optimized Models Trained on Both One-Hot and AA-Property Encoded Sequence Data

Name	Encoding Method	Top ML Algorithm	Grid-Search Optimized?	R^2 5% DPT	R^2 11% DPT	R^2 21% DPT	MAE 5% DPT	MAE 11% DPT	MAE 21% DPT
Wild-Types (WT)	One-Hot	XGB	N	0.884	0.844	0.820	11.1	13.6	15.9
			Y	0.900	0.864	0.844	10.8	13.0	14.9
	AA-Properties	GBR	N	0.871	0.860	0.812	11.7	13.6	16.0
			Y	0.905	0.888	0.861	10.2	12.0	13.5
Wild-Types MNM (WT-mnm)	One-Hot	XGB	N	0.933	0.916	0.846	9.84	10.8	12.2
			Y	0.948	0.924	0.864	8.41	10.0	11.2
	AA-Properties	GBR	N	0.941	0.921	0.858	9.32	10.5	11.4
			Y	0.950	0.930	0.885	8.19	9.58	10.3

We present R^2 and Mean Absolute Error (MAE) values for WT and WT-MNM models trained using 5%, 11%, and 21% Distance Percentile Thresholds (DPT). All R^2 and MAE values are calculated as the average of the values obtained from running PW-CV using 5, 8, 10, 12, 15, and 20 folds respectively.

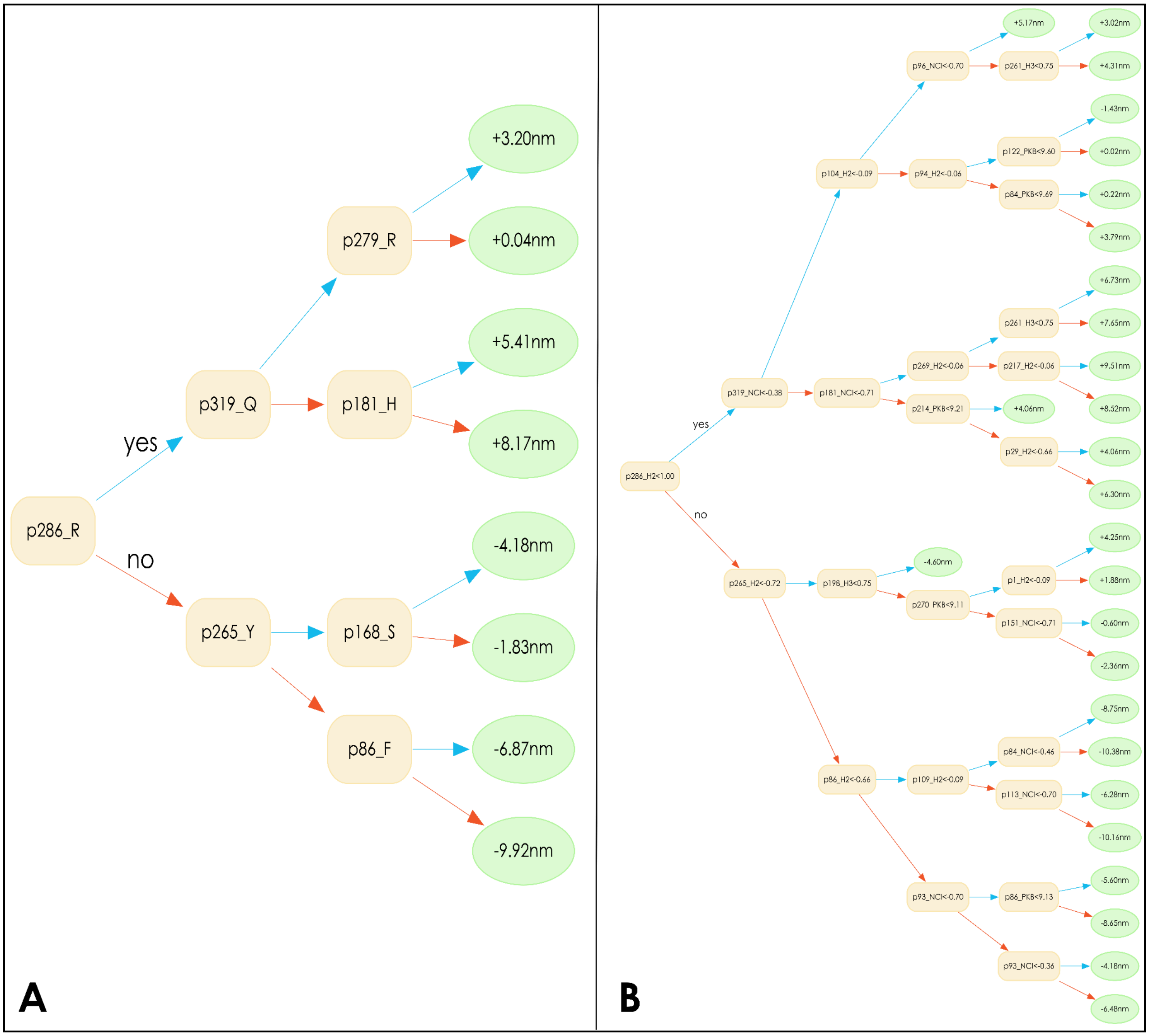

Supplementary Figure 1. (A) Decision-tree visualization from a singular decision tree of XGB model trained using One-Hot encoding on the WT dataset from VPOD_1.2 **(B)** Decision-tree visualization from a singular decision tree of XGB model trained using Amino-Acid Property encoding on the WT dataset from VPOD_1.2

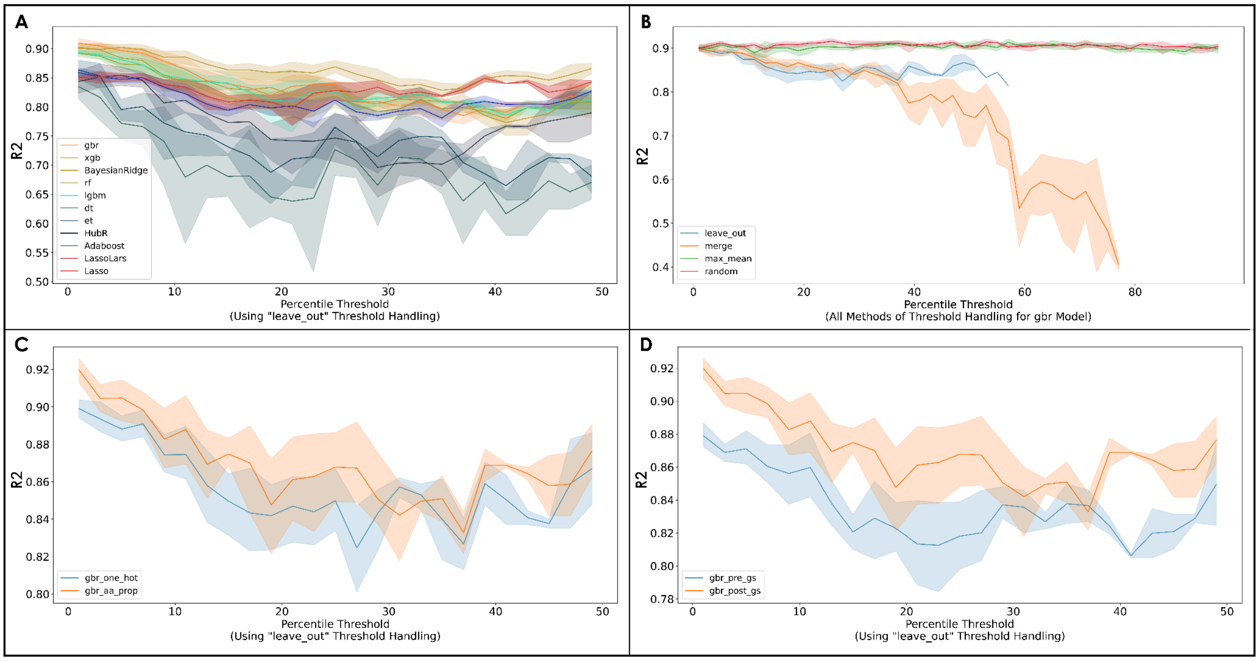

Supplementary Figure 2. Our analyses using *Phylogenetically-Weighted Cross-Validation* (PW-CV) demonstrated a clear relationship between the stringency of phylogenetic partitioning and model performance metrics. Generally, as the percentile-threshold increased, forcing greater phylogenetic distance between samples within cross-validation folds, we observed a decline in performance metrics such as R^2 for most algorithms tested. This trend suggests that standard cross-validation approaches, which do not account for phylogeny, may overestimate model generalizability due to the similarity between members of the training and testing splits. **(A-D)** Lighter color 'error-bars' represent the spread of model performances (R^2) relative to the number of cross-validation folds being assigned (5, 8, 10, 12, 15, and 20). **(A)** Line-graph comparison of change in various model R^2 trends relative to the change in phylogenetic distance threshold. As the threshold increases, the stringency for phylogenetic independence between members of the same cross-validation fold increases. The `'leave_out'` method shown here discards samples that violate the current phylogenetic distance threshold. (**B)** Line-graph comparing effects of relation-handling-method (RHM) choice on Gradient Boosted Regressor (GBR) model R^2 relative to the change in phylogenetic distance thresholds. The `'leave_out'` method stops at a percentile distance threshold of ~%60 because the resulting datasets created under those thresholds would be too small to train a functional model. For more information on all other RHMs, and more detail of the overall PW-CV pipeline, refer to Supplementary Methods (S3). **(C)** Line-graph comparing the effects of encoding methods used to train ML model (amino-acid properties (orange): gbr_aa_prop, or one-hot (blue): gbr_one_hot) on the change in GBR model R^2 relative to the change in phylogenetic distance thresholds. The superior performance of models trained with amino acid properties (aa-properties, orange) versus one-hot encoding (blue) is consistent across most phylogenetic distance thresholds. **(D)** Line-graph comparing the effects of 'pre' (blue) or 'post' (orange) grid-search optimization on the change in GBR model R^2 relative to the change in phylogenetic distance thresholds. The performance benefit of grid-search hyperparameter optimization ('post', orange) over default parameters ('pre', blue) persists even as the stringency of PW-CV increases.

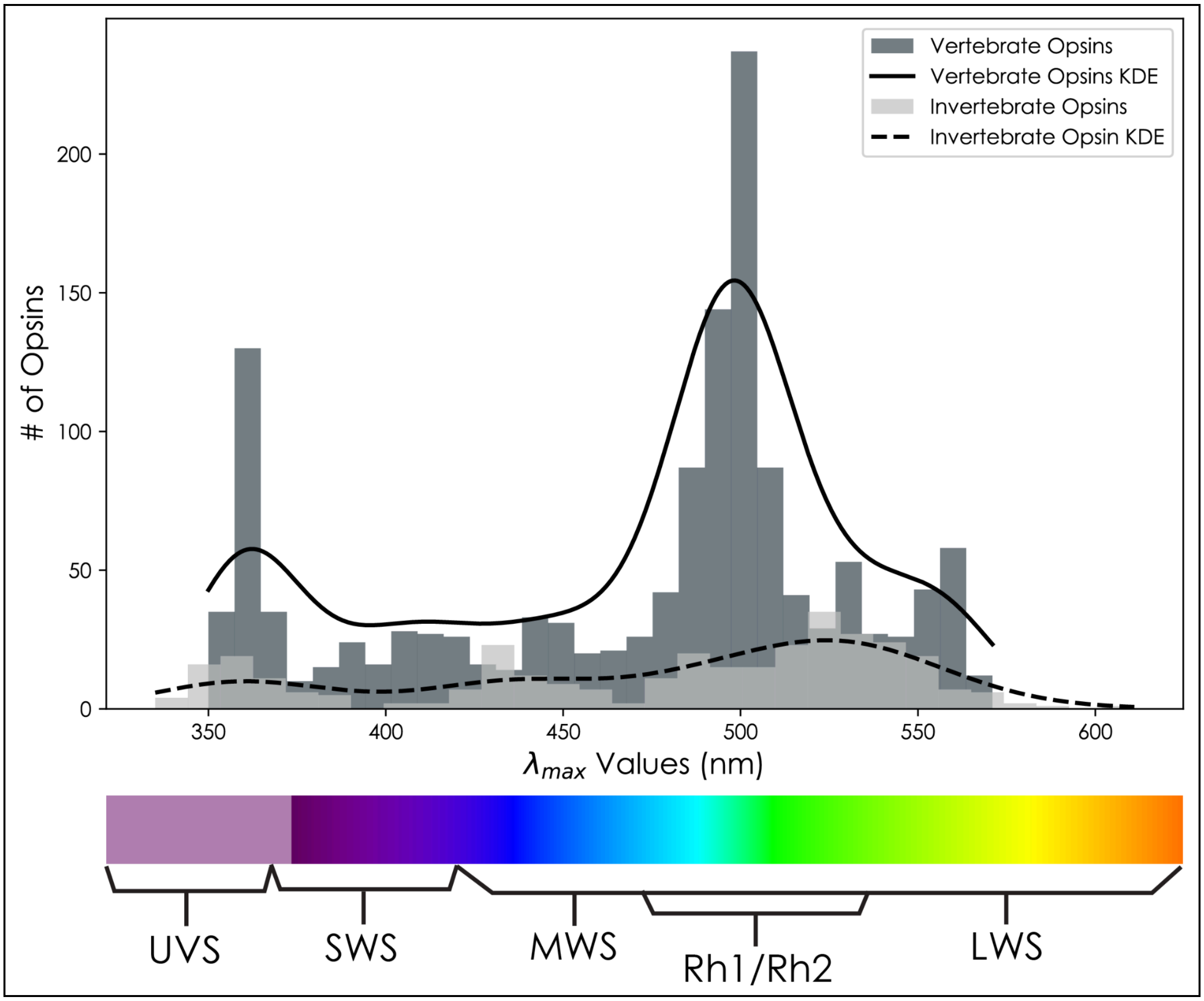

Supplementary Figure 3. Histogram distributions of vertebrate and invertebrate opsins and absorbance data—λ_{max}—from *VPOD_1.3* with a scaled kernel density estimate (KDE) curves overlaid to better visualize the general shape and characteristics of our λ_{max} distributions. Note an obvious data bias for vertebrate opsins, especially those with λ_{max} values between 350–375 nm and 480–510 nm, probably due to focal research on UVS and Rh1 opsins.

Supplementary Data 1: Fasta File for *C. phantasticus* LWS paralogs

```
>C_phantasticus_LWS1
MAEDWGSVVFAARRRNEEETTRESIFVYTNSNNTRDPFDGPNYHIAPRWVYNIATVWMFVVVI
LSTFTNGLVLVATAKFKKLRHPLNWILVNLAVADIAETLIASTISVVNQIYGYFILGHPLCIIEGYTVA
ACGIAGLWSLTVISWERWIVVCKPFGNVKFDEKWATAGIIFTWVWSLFWCAPPIFGWSRYWPH
GLKTSCGPDVFSGNEDPGVRSYMVTLMLTCCILPLSVIIFCYICVWMAIRAVAQQQKDSESTQKA
EKEVSRMVVVMILAYCFCWGPYTFFVCFGTAYPGYAFHPLAAAMPAYFAKSATIYNPIIYVFMNR
QFRVCIYQMFGKKVDDGSEVSSTSKTEVSSVAPA
>C_phantasticus_LWS2
MADRVMFSARRHHEEETTRASIFTYTNNNNTRDPFVGPNYDIAPRWVYNACTVWMIFVVIASLF
TNGLVLVATAKFKKLRHPLNWILVNLAVADIGETILASTISIINQIFGYFILGHPMCIFEGYVVSVCGI
AGLWSLTVISWERWVVVCKPFGNVRFDGKWATAGIIFSWIWAILWCAPPIFGWSRYWPHGLKT
SCGPDVFSGSEDPGVKSYMITLLFTCCILPLAVIIICYIFVWSAIHSVAQQQKDSESTQKAEREVT
RMVIVMVVAFVICWGPYATFATFSACNPGYAWHPLTASLPAYFAKGATIYNPIIYVFMNRQFRNCI
LQLFGKKVEDGSEITSSSTTEVSTAS
```

Supplemental Methods

S1. OPTICS Implementation and Availability

OPTICS_v1.2 was implemented in Python (v3.11.9) and relies on libraries including *Scikit-learn* and *BioPython* (see *requirements.txt* on the OPTICS GitHub). External dependencies are NCBI BLAST+ (Camacho et al. 2009) and MAFFT (Katoh and Standley 2013); MAFFT is bundled for Windows. Available models in *OPTICS_v1.2* (and their command-line references) include those trained on WDS (*whole-dataset*), WT (*wildtype*), Vert (*vertebrate*), WT-Vert (*wildtype-vert*), Invert (*invertebrate*), and T1 (*type-one*) datasets. OPTICS uses *Joblib* and *Multiprocessing* for parallelized predictions. Output directories are standardized as *optics_on_{output_dir}_{timestamp}*. Source code is available at GitHub (https://github.com/VisualPhysiologyDB/optics) under GPL v3. A web version is hosted on a Galaxy Platform server (Galaxy Community 2024)[http://galaxy-dev.cnsi.ucsb.edu:8080/?tool_id=optics_1&version=latest]. OPTICS is an ongoing project and may accumulate more features than originally described in this publication.

S2. Bootstrapping Pipeline Details

Bootstrap ensembles were generated for WDS, WT, Vert, Invert, and WT-Vert VPOD data subsets. Individual bootstrapped models were saved as PKL files (e.g., *gbr_69.pkl*) in dataset-specific folders (e.g., *wds_bootstrap*). Scripts used to generate ensembles are in

scripts_n_notebooks/vpod_ML_workflows/bootstrap_model_gen on the VPOD GitHub repository.

S3. Physicochemical amino-acidProperties

We selected twelve physicochemical properties of amino acids (aa-properties): hydrophobicity (H1), hydrophilicity (H2), hydrogen bonding (H3), polarity (P1), polarizability (P2), volume (V), net charge index of side chains (NCI), average mass of the amino-acid(MASS), solvent-accessible surface area (SASA), acid-dissociation constant (PkA), base-dissociation constant (PkB), and side-chain type (SCT). Ten of these were used in previous studies (Li et al. 2016; Inoue et al. 2021), PkA and PkB were sourced from *MilliporeSigma* reference charts, and SCT was a categorical property we defined. All properties, except for SCT, were normalized (0-1 or -1-1) in-line with a similar implementation of this methodology (Li et al. 2016)(Supplementary Table 1). The *AminoAcidPropertyEncoder* function was integrated into the *deepBreaks* pipeline (Baghbanzadeh et al. 2023; Frazer et al. 2024). Helper functions *aaprop_importance_from_pipe* and *dp_aa_prop_plot* were written for visualizing site-wise property importance and relationships with λ_{max} , respectively. These functions are available in *preprocessing.py* and *visualization.py* scripts within the *deepBreaks* folder in our VPOD GitHub repository (*scripts-n-notebooks/vpod_ML_workflows/vpod_scripts*). The combinatorial aa-property encoding experiment workflow, *aa_prop_combos.py*, can be found under *scripts-n-notebooks/vpod_ML_workflows/subtests* in VPOD.

S4. Phylogenetically-Weighted Cross-Validation (PW-CV) Details

Purpose and Tree Construction: To reduce inflated metrics in ML models caused by phylogenetic non-independence, we developed 'Phylogenetically-Weighted Cross-Validation' (PW-CV). PW-CV assigns genes to cross-validation folds such that each fold contains phylogenetically dissimilar sequences, mitigating the overestimation of accuracy that occurs when closely related sequences appear in both training and testing sets. We first created a pairwise phylogenetic distance matrix for all target opsins from a maximum likelihood gene tree estimated with IQ-TREE (Minh et al. 2020). We used the 'LG+F+R7' model for constructing the WT opsin gene tree, as determined by IQ-Tree's 'ModelFinder Plus' (MFP+LM) parameter, and used 1000 ultra-fast bootstrap replicates to assess branch support.

Fold Initialization and Distance Thresholds: PW-CV initializes folds by selecting the *n* most mutually distant genes, where *n* is the user-specified number of folds. Remaining genes are iteratively added to the fold whose members are most phylogenetically dissimilar, provided the gene exceeds a minimum distance threshold. This threshold is defined by a user-specified percentile of all pairwise distances in the tree (e.g., if the percentile is set to 5%, the threshold is the distance at the 5th percentile of all pairwise distances).

Relation-Handling-Methods (RHMs): To manage tips that fell below the distance threshold for all available folds, we defined four 'relation-handling-methods' (RHMs):

- '`random`': Assigns a tip violating the distance-percentile-threshold to a random fold.
- '`merge`': Assigns the tip to the fold containing the member to which it is most closely related. In practice, this method yielded highly variable outcomes and sometimes underestimated performance due to the creation of unbalanced folds.
- '`max_mean`': Assigns the tip to the fold with the greatest mean phylogenetic distance from it.
- '`leave_out`': Assigns a fold identity of '-1' to the tip, which is later excluded from training. This provided the strictest phylogenetic separation and the most conservative evaluation of model performance, but it led to a consistent reduction in the size of the training datasets resulting in lower absolute performance than the 'random' or 'max_mean' methods.

Extended Results: Using this pipeline, we systematically varied the percentile threshold (1–95%), number of folds (5–20), and RHMs to assess their impacts on model performance. As thresholds became more stringent and forced greater phylogenetic distance, R^2 generally declined across most algorithms, confirming that standard k-fold cross-validation overestimates generalizability when ignoring phylogenetic relationships. Changing the number of folds had much smaller effects on performance than adjusting the distance thresholds. Under strict phylogenetic partitioning, Gradient Boosted Regressor (GBR), Extreme Gradient Boosted Regressor (XGB), and Random Forest Regressor (RF) consistently ranked as top performers, whereas Lasso, HuberRegressor, and ExtraTrees performed the worst. Optimization of hyperparameters by grid-search consistently improved performance under PW-CV.

Extended Discussion: Also important to reliability is evaluating models in a way that accounts for evolutionary non-independence. Standard cross-validation often inflates performance because closely related sequences can appear in both training and test sets (Roberts et al. 2017). Our phylogenetically weighted cross-validation (PW-CV) examines this bias by incorporating sequence-based phylogenetic distances into the partitioning process. As phylogenetic separation increases, performance metrics generally decline, yielding more conservative estimates of generalization to novel sequences. Importantly, there is no universally optimal phylogenetic threshold because higher thresholds exclude more closely related sequences, increasing conservatism, but eventually reduce the dataset to the point where accuracy declines because less training data are available. This makes the selection of a single "best" threshold inherently arbitrary. Instead of a single threshold, researchers could report performance across a range of thresholds, or examine overall trends. For example, large early drops in performance at low thresholds may indicate a phylogenetically imbalanced dataset in which many closely related genes are rapidly removed.

Code Availability: All scripts for PW-CV are located in '*scripts_n_notebooks/vpod_ML_workflows/subtests/phylo_weighted_cv'* and results are in '*result_files/phylo_weighted_cv'* on the VPOD GitHub repository.

S5. Hyperparameter Optimization (Grid Search) Details

The full set of ML algorithm-specific hyperparameters explored during grid search can be found in the *get_exp_params* module in *deepBreaks/utils_alt.py* on the VPOD GitHub repository. For one-hot encoded models, results are in Supplementary Table 3. For aa-property encoded models, the top five combinations by R^2 and top five by MAE from the combinatorial analysis were subjected to grid search; final results are in Supplementary Table 4. Scripts (*vpod_one_hot_grid_search_iter.py* and *vpod_aa_prop_grid_search_iter.py*) are in *scripts_n_notebooks/vpod_ML_workflows/subtests/grid_search* in VPOD.

S6. Mine-N-Match (MNM) Pipeline Details

VPOD_in_vivo_v1.0: The individual components of *VPOD_in_vivo_v1.0* were sourced from the *Longcore (Longcore 2023)*, *Murphy_Westerman (Murphy and Westerman 2022)*, *Caves_Fish (Schweikert et al. 2019), Porter_1 (Porter 2005), Porter_2 (Porter et al. 2007)*, and *J_Kooi (van der Kooi et al. 2021)* publications; in combination with our own non-exhaustive in-house dataset. Our in-house dataset of *in-vivo* λ_{max} data was created following similar methods in Frazer et al. (2024), cataloging species, phylum, λ_{max} , error, cell type, chromophore, life-stage, and literature source (linked to VPOD *litsearch* table) in *VPOD_in_vivo_data.csv.* However, one should note that this custom data-set was not originally compiled for the purpose of this study; rather we repurposed this data in an *ad-hoc* manner. As such, we do not label this dataset as an independent element of VPOD which can be versioned in the same way as our heterologous datasets. We used the *merge_accessory_dbs* function in *mine_n_match_functions.py* (located under the directory *scripts_n_notebooks/vpod_ML_workflows/mine_n_match/mnm_scripts* in VPOD) to harmonize these data collections into a single compendium. We deemed this resulting compendium of *in-vivo* λ_{max} data, *VPOD_in_vivo_v1.0* (which can be found under the directory *scripts_n_notebooks/vpod_ML_workflows/mine_n_match/data_sources/lmax/VPOD_in_vivo_1.0_2025-09-22_15-52-28.csv*)

Genotype Mining: We queried the NCBI taxonomy database. The standardized NCBI query for opsin coding sequences is detailed in *mine_n_match_functions.ncbi_fetch_opsins*. Mined NCBI data was saved as *mnm_on_all_dbs_ncbi_q_data_cleaned.csv* and *mined_mnm_on_all_dbs_cleaned.fasta.* Instances where we received opsin data from species not part of the query were marked as unintended hits and isolated them for future manual inspection. This data was saved to *mnm_on_all_dbs_ncbi_q_potential_hits.csv*, and species which yielded no opsin data from our queries were saved to *species_w_no_hits.txt*. Merged NCBI and

accessory data were saved to *mined_and_acc_seqs.csv* and *mined_and_acc_seqs.fasta*. All data from this process are under the report directory *scripts_n_notebooks/vpod_ML_workflows/mine_n_match/mnm_data/mnm_on_all_dbs_2025-10-03_21-17-52* in VPOD.

Matching Process: Sequences flagged by OPTICS as '*blastp unsuccessful'* were dropped before matching. Sequences with 100% identity to existing VPOD entries or with a difference >10 nm between predicted and *in-vivo* λ_{max} were dropped after matching. The final MNM dataframe was saved as *mnm_on_vpod_in_vivo_results_fully_filtered.csv* (which can be found under the directory *scripts_n_notebooks/vpod_ML_workflows/mine_n_match/mnm_data/mnm_on_all_dbs_2025-10-03_21-17-52*). The MNM workflow, *mine_n_match-wf.ipynb*, is under the directory *scripts_n_notebooks/vpod_ML_workflows/mine_n_match* in VPOD.

S7. VPOD_v1.3 Integration and OPTICS_v1.3 Release

VPOD_v1.3 sequences and metadata were formatted as per Frazer et al. (2024). The five new models (*whole-dataset-mnm*, *wildtype-mnm*, *vertebrate-mnm*, *wildtype-vert-mnm*, *invertebrate-mnm*) and their bootstrap versions are available in *OPTICS_v1.3* on the OPTICS GitHub repository (https://github.com/VisualPhysiologyDB/optics).
